# Improvement of the thermal stability of dendritic silver-coated copper microparticles by surface modification based on molecular self-assembly

**DOI:** 10.1186/s40580-021-00265-8

**Published:** 2021-05-20

**Authors:** Tae Hyeong Kim, Hyeji Kim, Hyo Jun Jang, Nara Lee, Kwang Hyun Nam, Dae-won Chung, Seunghyun Lee

**Affiliations:** 1 Department of Chemistry, Gachon University, Seongnam-Si, 13120 Republic of Korea; 2 Department of Polymer Engineering, College of Engineering, University of Suwon, Suwon, 18323 Republic of Korea; 3 Mazal Co., Ltd, #505, 506, Business Incubation Center, Kyunggi University, Suwon, 16227 South Korea; 4 Department of Chemical and Molecular Engineering, Hanyang University, Ansan-Si, 15588 Republic of Korea; 5 Center for Bionano Intelligence Education and Research, Hanyang University, Ansan-Si, 15588 Republic of Korea

**Keywords:** Ag/Cu, Alkanethiol, Conductive film, Thermal stability

## Abstract

In the study reported herein, silver-coated copper (Ag/Cu) powder was modified with alkanethiols featuring alkyl chains of different lengths, namely butyl, octyl, and dodecyl, to improve its thermal stability. The modification of the Ag/Cu powders with adsorbed alkanethiols was confirmed by scanning electron microscopy with energy dispersive spectroscopy, X-ray photoelectron spectroscopy, and thermogravimetric analysis. Each powder was combined with an epoxy resin to prepare an electrically conductive film. The results confirmed that the thermal stability of the films containing alkanethiol-modified Ag/Cu powders is superior to that of the film containing untreated Ag/Cu powder. The longer the alkyl group in the alkanethiol-modified Ag/Cu powder, the higher the initial resistance of the corresponding electrically conductive film and the lower the increase in resistance induced by heat treatment.

## Introduction

Electrically conductive adhesives are widely used in the fabrication of electronic devices and mechanical attachments, and generally consist of a polymer matrix and conductive metal fillers. The purpose and application range of electrically conductive adhesives have expanded dramatically over time and various materials, such as metals (including Au, Ag, Al, and Cu [1, 2]), carbon fibers [3], and graphite [4, 5] have been employed as fillers. While noble metals are chemically stable and demonstrate excellent electrical and thermal properties, Cu and Ni are researched and applied more extensively than noble metals owing to their greater affordability. However, Cu is more widely used than Ni, despite its vulnerability to oxidation, since the electrical properties of Ni are inferior to those of Cu. Multiple studies have explored strategies to prevent or inhibit the corrosion of Cu in the presence of oxygen. Strategies that have been developed to prevent the oxidation of Cu, including the employment of protective azole compounds [6, 7], amines [8], and self-assembling thiol compounds [9], are well organized in a published review [10].

Ag-coated Cu (Ag/Cu) conductive metal powders are commonly used in industry and while various methods exist to produce these Ag-coated Cu powders [11, 12], related technologies are typically protected by patents [13–16]. The vulnerability of Ag/Cu powders to oxidation depends on their Ag content. For example, the electrical performance of an electrically conductive, epoxy-based adhesive containing 20 vol% Ag/Cu powder with a Ag content of 15 % remains relatively steady for approximately 2 h at 200 °C, however, its resistance increases rapidly above 250 °C [17]. In contrast, the resistance of an adhesive containing non-coated Cu powder increases significantly at a mere 100 °C. Studies have revealed that the electrical properties of a Cu-containing electrically conductive adhesive change under pressure [18] and the distribution of Ag on the surface of Ag-coated Cu particles, determined by the Ag content and coating process, directly affects the degree of oxidation observed [19]. Ag/Cu powders are widely applied in electrically conductive adhesives and it is possible to fabricate Ag/Cu powders with different shapes through various manufacturing methods [12, 20–22]. It is well known that spherical Ag/Cu powder particles are suitable for anisotropic conductive adhesives, and Ag/Cu flakes are suitable for isotropic conductive adhesives [23, 24]. It has also been reported that Ag/Cu powders treated with silane coupling agents show more reliable electrical properties [24]. These research results have informed the analytical and chemical study of the behavior of Ag/Cu powder in epoxy-based adhesives [25].

In this study, the surfaces of dendritic Ag/Cu powders were modified with self-assembling alkanethiols. These modified surfaces were analyzed by scanning electron microscopy with energy dispersive X-ray spectroscopy (SEM-EDS), X-ray photoelectron spectroscopy (XPS), and thermogravimetric analysis (TGA). In addition, epoxy-based composite films containing the Ag/Cu powders modified with alkanethiols featuring different alkyl-chain lengths were prepared to investigate their thermal stability and electrical resistance.

## Experimental

### Chemicals

The alkanethiols (butanethiol, octanethiol, and dodecanethiol) used to modify the surfaces of Ag/Cu powders were purchased from Sigma-Aldrich. Ag/Cu powder dispersed in isopropyl alcohol (IPA), without surface treatment after Ag coating, was supplied by JC Metal (Korea). The epoxy resin (YDPN 644, epoxy equivalent weight = 195–235 g/eq) and curing agent, phthalic anhydride (MNA), employed in the preparation of electrically conductive films were supplied by Kukdo Chemical (Korea). Toluene (99.5 %, Daejung Chemical, Korea) served as a diluent. All chemicals were used as received, without purification.

### Surface modification of Ag/Cu powders

To prepare octanethiol-modified Ag/Cu (Ag/Cu-C_8_) powder, 1 g of Ag/Cu powder was placed in a conical tube and dispersed in 30 ml of anhydrous ethanol using a vortex mixer. After centrifuging the mixture at 6000 rpm for 10 min, the supernatant was decanted and the precipitate was washed with anhydrous ethanol. The procedure was repeated three times. Next, 40 mL of an ethanol solution containing octanethiol (50 mM) was added to the conical tube and dispersed well using a vortex mixer for a few tens of seconds. The reagents were allowed to react while mixing in a vertical rotary tube mixer (IKA Trayster Rotator, 50 rpm) for 24 h. After thorough dispersion using an ultrasonicator and a vortex mixer, centrifugation was performed 5 times (each time the mixture was centrifuged at 6000 rpm for 10 min and the supernatant decanted before the addition of 40 mL anhydrous ethanol) to remove unreacted octanethiol. Finally, the precipitate was vacuum dried at 40 °C to obtain the modified Ag/Cu powder. Butanethiol and dodecanethiol were reacted with Ag/Cu powder to prepare Ag/Cu-C_4_ and Ag/Cu-C_12_ powders, respectively, using the same surface modification procedure.

### Preparation and evaluation of electrically conductive films

Each modified Ag/Cu powder (6.2 g) was mixed with epoxy resin (2 g), curing agent, and toluene (5 g), and stirred at 500 rpm for 2 min. Each mixture was defoamed for 3 min under vacuum to prepare a conductive paste that was repeatedly applied (10 times) onto a polyimide film using a Mayer rod coater (wet film thickness of 22.9 μm) and dried at 100 °C for 2 min. The prepared films were cured at 160 °C for 1 h, and cut to an appropriate size (7 cm × 1 cm). The surface roughness at five points on each film (the center point was 1 cm from the top) was measured to obtain the average value and standard deviation. The thermal properties of the conductive films were examined using two methods. As a simple test, the change in the linear resistance of each electrically conductive film over a length of 5 cm was measured in an oven at 180 °C over time. The conductive films were cured and laminated on a test circuit coupon at 160 °C under a pressure of 10 kgf/cm^2^ for 1 h. The initial linear resistance of each film was measured over a distance of 5 cm before the specimens were placed in a solder pot at 260 °C for 10 s. After cooling at 25 °C for 10 min, the linear resistance was again measured over a distance of 5 cm. This heat treatment and linear resistance measurement process was repeated three times.

### **Characterization of alkanethiol-modified Ag/Cu powder surfaces**

The morphological and chemical analyses of the modified Ag/Cu powders were performed by SEM-EDS (Apreo, FEI), XPS (K-Alpha, Thermo Fisher Scientific), and TGA (TGA 4000, Perkin Elmer). The TGA was carried out under a nitrogen atmosphere (nitrogen flow rate: 50 mL/min) while the temperature was increased from room temperature to 600 °C at a rate of 10 °C/min. To evaluate the oxidation behavior of the Ag/Cu particles, TGA was performed under the same conditions as above without nitrogen injection. The surface roughness values (arithmetic mean roughness (Ra), ten-point mean roughness (Rz)) of the films ​​were measured to investigate the dispersion of the Ag/Cu particles in the epoxy resin using a surface roughness tester (Mitutoyo Surftest, Japan).

## Results and discussion

### Alkanethiol modification of Ag/Cu surfaces and their characterization

**Fig. 1:**
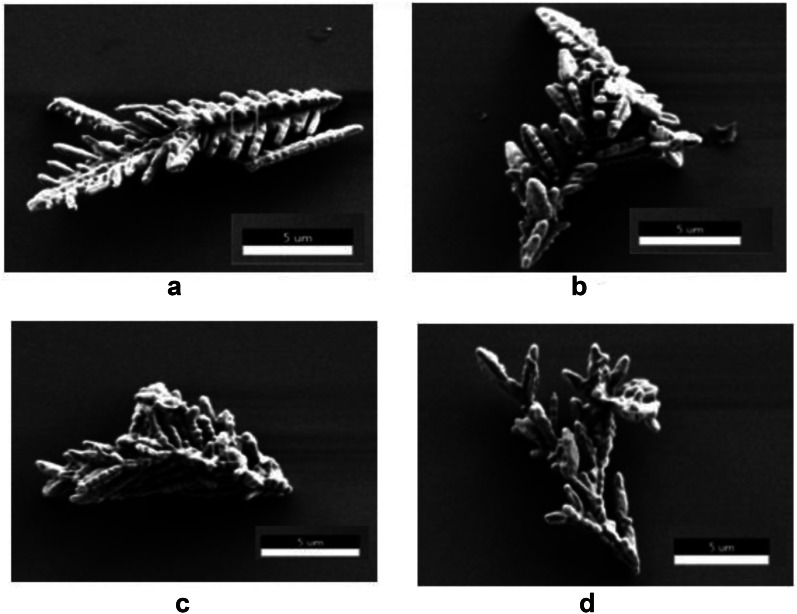
SEM images of **a** untreated Ag/Cu, **b** Ag/Cu-C_4_, **c** Ag/Cu-C_8_, and **d** Ag/Cu-C_12_ powder particles

The alkanethiol modification of the Ag/Cu powder particle surfaces in this study is similar to a previously reported method of Cu particle surface modification through self-assembling thiol compounds [9]. The surface morphology of the modified Ag/Cu powder particles was investigated by SEM. Figure 1 shows SEM images of dendritic Ag/Cu powder particles that are untreated and modified with different alkanethiols, respectively. The Ag/Cu powder particle in Fig. 1a has various dendritic protrusions on its surface and its major axis is approximately 15–20 μm long. The alkanethiol-modified Ag/Cu powder particles do not differ significantly in shape or size from the untreated Ag/Cu powder particle. The surfaces of the protrusions on the alkanethiol-modified Ag/Cu powder particles seem slightly less rough than those of the protrusions of the untreated Ag/Cu powder particle, however, it is difficult to confirm the surface modification through SEM alone. Therefore, EDS and XPS analyses were performed to compare the relative constituent element contents based on the chemical compositions of the surface-modified Ag/Cu powder particles; the results are summarized in Table 1.

**Table 1:** Relative atomic contents (%) of Ag/Cu and modified Ag/Cu particles measured by EDS and XPS

	Relative atomic % (EDS)				Relative atomic % (XPS)			
	Ag/Cu	Ag/Cu-C_4_	Ag/Cu-C_8_	Ag/Cu-C_12_	Ag/Cu	Ag/Cu-C_4_	Ag/Cu-C_8_	Ag/Cu-C_12_
C/Ag	1.89	5.51	5.97	5.29	1.25	6.25	7.34	8.16
S/Ag	0.01	0.31	0.14	0.08	0.04	1.12	0.83	0.73
S/C	0.00	0.06	0.02	0.01	0.03	0.16	0.13	0.09

The EDS data in Table 1 show that the S content of the untreated Ag/Cu powder (relative to its Ag (S/Ag) and C (S/C) contents) is negligible; unlike the relative S contents of the alkanethiol-modified Ag/Cu powders. A similar trend is observed in the relative C (C/Ag) contents of the untreated and alkanethiol-modified Ag/Cu powders, confirming that the surfaces of the treated Ag/Cu powders are modified by alkanethiol compounds.

**Fig. 2:**
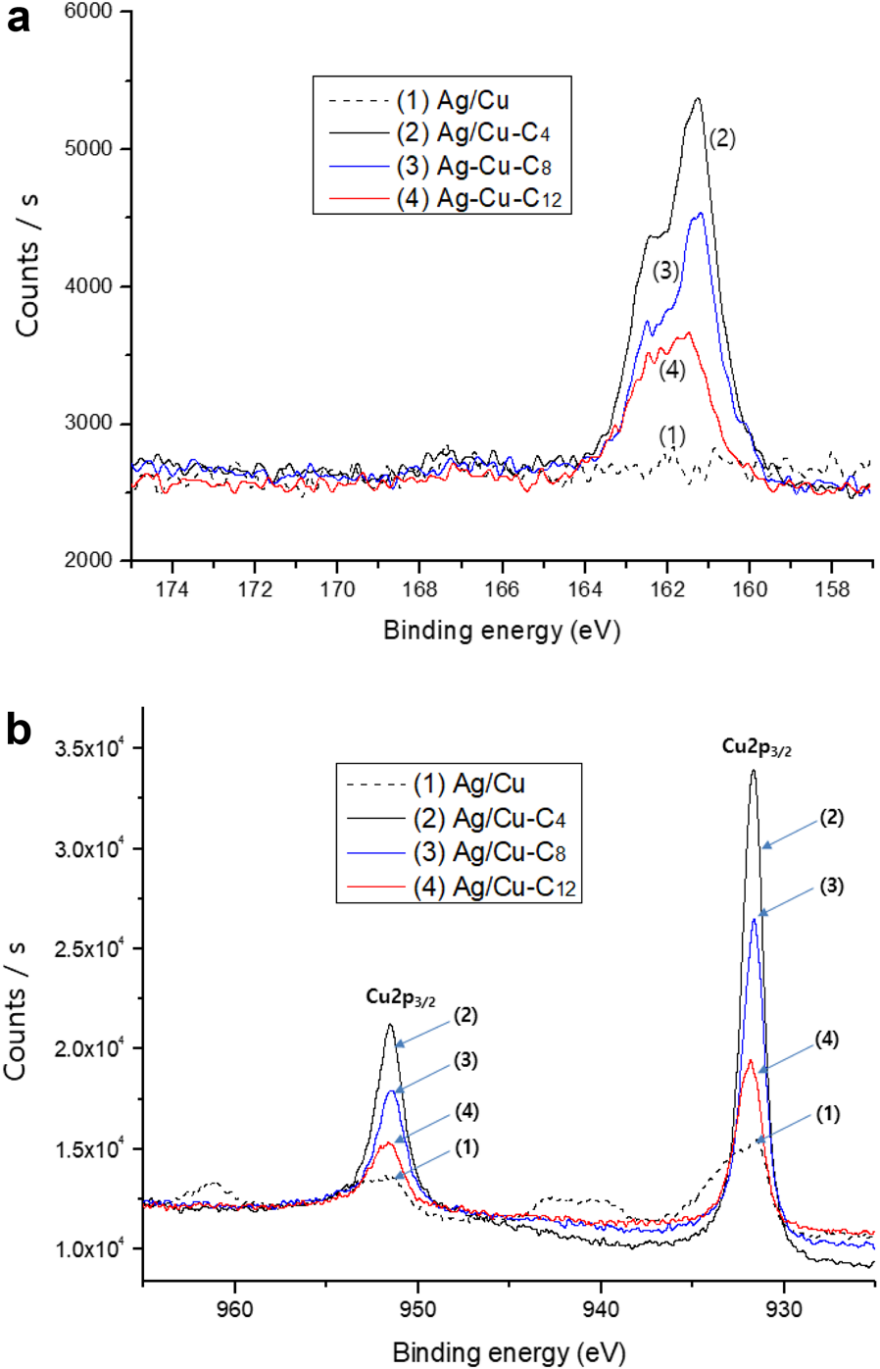
High-resolution XPS spectra reflecting the **a** S2p and **b** Cu2p peaks of (1) Ag/Cu, (2) Ag/Cu-C_4_, (3) Ag/Cu-C_8_, and (4) Ag/Cu-C_12_ powders

The C content of the alkanethiol-modified Ag/Cu powders (relative to their Ag (C/Ag) content) increases with increasing alkyl-chain length, whereas their relative S content decreases with increasing alkyl-chain length. These observations are explained by the structures of the alkanethiols, i.e., the longer the alkyl chain, the smaller the atomic ratio of S to C. The decreasing S/C values reflected in Table 1 clearly illustrates this phenomenon. Furthermore, the trends observed in the results obtained by XPS are similar to those observed in the results obtained by EDS despite the fact that the results of the two analytical methods do not correspond quantitatively because they differ in principle. A comparison of the XPS spectra of the untreated and alkanethiol-modified Ag/Cu powders in Fig. 2a reveals that a S2p peak, indicating the presence of S, does not appear in the spectrum of the untreated Ag/Cu powder, whereas S2p peaks at 161.8 eV are observed in the spectra of the alkanethiol-modified Ag/Cu powders. The intensity of the S2p peak decreases with increasing alkyl-chain length of the alkanethiol-modified Ag/Cu powders. The absolute areas under the various peaks in the spectra of the specimens cannot be quantitatively compared, but the results shown in Fig. 2 are consistent with the relative atomic contents summarized in Table 1. The XPS spectrum of the untreated Ag/Cu powder (1) in Fig. 2b shows four typical peaks associated with partially oxidized Cu [26]. However, in the spectra of the alkanethiol-modified Ag/Cu powders, only the Cu2p_3/2_ and Cu2p_1/2_ peaks at 931.8 and 952.5 eV are observed, which suggest that the presence of Cu oxide is limited by the adsorbed alkanethiols on the Ag/Cu particle surfaces. In fact, in another study of Cu particles modified with dodecanethiol, the simultaneous analysis of multi-element XPS spectra revealed peaks attributed to Cu2p_3/2_ and Cu2p_1/2_ at 931.9 and 952.2 eV, respectively [27]. The relative constituent element contents of the alkanethiol-treated and untreated Ag/Cu powders determined by EDS and XPS suggest that the surfaces of the treated Ag/Cu powders are well modified with alkanethiols, and the amount of C can be controlled by changing the alkyl-chain lengths of the alkanethiols. Moreover, the alkanethiols are well self-assembled on the surfaces of the treated Ag/Cu powders based on the S and Cu peaks observed in their XPS spectra.

**Fig. 3:**
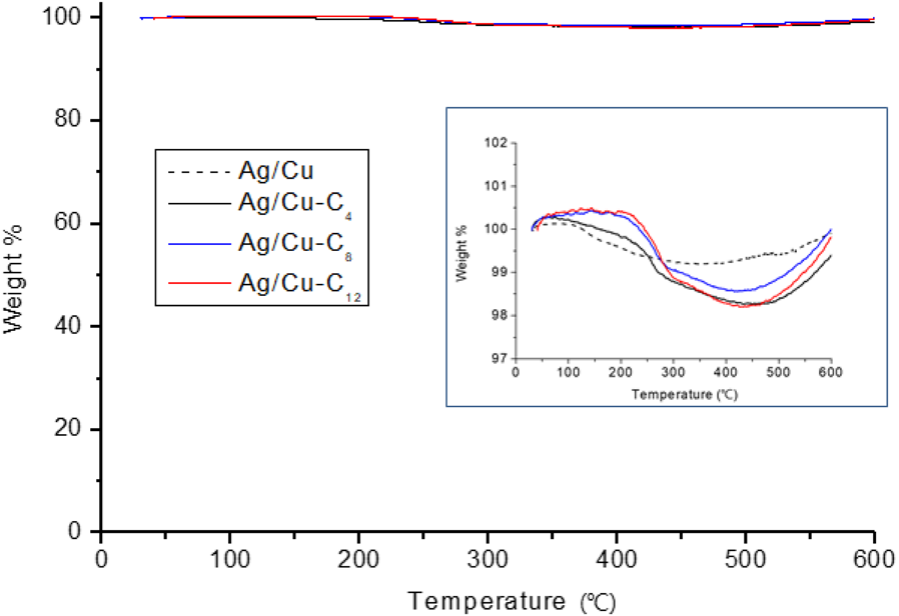
TGA of untreated Ag/Cu and alkanethiol-modified Ag/Cu powders under nitrogen atmosphere

In general, when the surfaces of metals or inorganic materials are functionalized or modified with organic materials, TGA enables the measurement of the organic material content by monitoring the weight loss of a relevant sample as a function of temperature or time [28]. In this study, we evaluated the weight loss as a function of temperature under a nitrogen atmosphere. The TGA curves in Fig. 3 suggest that there is almost no change in the weight of the samples with increasing temperature and no significant difference is observed between the TGA curves of the untreated and alkanethiol-modified Ag/Cu powders, even in the inset in Fig. 3 that magnifies the region of change.

**Fig. 4:**
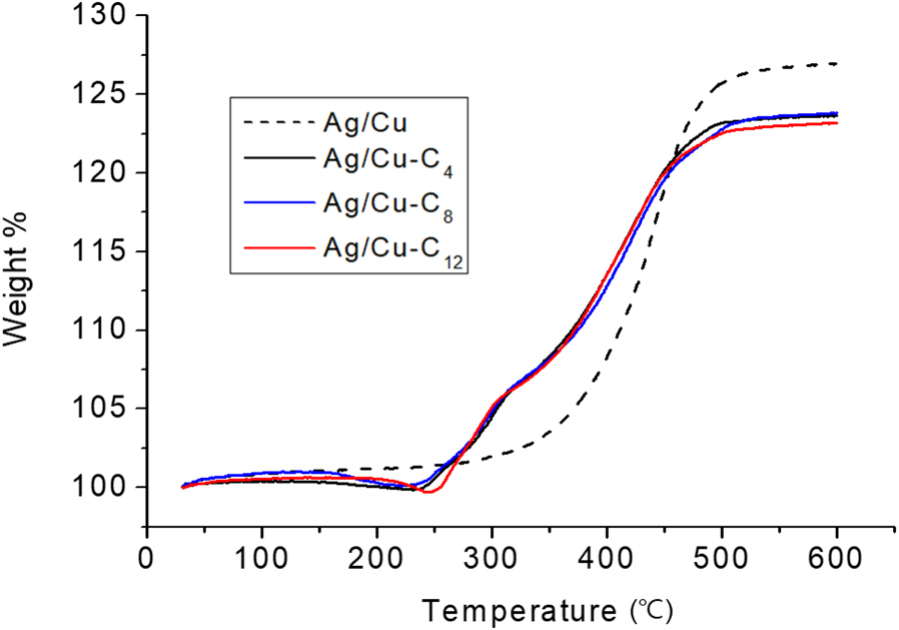
TGA of untreated Ag/Cu and alkanethiol-modified Ag/Cu powders without nitrogen injection

The greatest difference between the maximum and minimum recorded weight percentages is approximately 2 wt%; therefore, it is reasonable to conclude that the content of the alkanethiols bound to the surfaces of the treated Ag/Cu powders is 2 wt% or less. The dendritic Ag/Cu powder particles used in this study have a few micron scale and high density; therefore, the alkanethiols adsorbed to the surface of a treated Ag/Cu powder particle does not significant contribute to its weight. In practice, the TGA of Ag/Cu powders evaluates their degree of oxidation at high temperatures rather than their organic matter content [12, 17, 29]. Accordingly, the degree of oxidation of the Ag/Cu powders was examined by TGA under the same conditions but without nitrogen injection. As shown in Fig. 4, in the presence of oxygen, the weight of every sample increases with increasing temperature, indicating the oxidation of Cu. A previous study featuring surface-untreated Ag/Cu powders, found that the weight of the Ag/Cu powder starts to increase once the temperature reaches ~ 250 °C and at 400 °C the weight has increased by ~ 9 wt% [26]. Another study reported that limited oxidation occurs from 175 °C, and between 175 and 260 °C the weight increases by ~ 1 wt%. At temperatures exceeding 260 °C, oxidization occurs more rapidly and a weight increase of ~ 7 wt% is recorded once the temperature reaches 400 °C [17]. In this study, the weight of the untreated Ag/Cu powder increases very slowly from ~ 170 °C; however, the rate of weight increase accelerates from ~ 260 °C and a weight increase of approximately 7.5 wt% is recorded once the temperature reaches 400 °C. This result is consistent with the results reported by previous studies. In contrast, in the presence of oxygen, the weights of alkanethiol-modified Ag/Cu powders decrease by ~ 1 wt% up to 250 °C before increasing. It is considered that the initial slight decrease in weight is caused by the reduction of the Ag/Cu surface upon the adsorption of alkanethiols; the subsequent weight gain observed for the alkanethiol-modified Ag/Cu powders is attributed to the oxidation of the metal surfaces in the presence of oxygen. The difference in the weight gain behaviors of the respective alkanethiol-modified Ag/Cu powders is negligible.

### Evaluation of electrically conductive films containing alkanethiol-modified Ag/Cu powders

After electrically conductive films were prepared by dispersing the various Ag/Cu powders in epoxy resin, the degree of dispersion of the alkanethiol-modified Ag/Cu powders was indirectly evaluated by measuring the surface roughness. While there are various ways of characterizing surface roughness, it is most commonly expressed in terms of the Ra and Rz. Ra is defined as the arithmetic mean of the absolute values of the ordinates of the measured profile relative to the midline along the measurement length. Rz is defined as the mean difference between the 5 lowest minima and the 5 highest maxima of the assessed surface profile [30]. In this study, the Ra and Rz values at five locations on each of the 7 cm × 1 cm films were measured; their mean and standard deviation are summarized in Table 2. The dispersion of the hydrophobic silica in the film containing untreated Ag/Cu powder is poor, which is easily confirmed visually, and its surface roughness is relatively high based on its Ra and Rz values of ~ 2.403 and ~ 13.459 μm, respectively. The Ra and Rz values of the films containing alkanethiol-modified Ag/Cu powders of 0.6–0.7 and 7–8 μm, respectively, are significantly lower than those of the film containing untreated Ag/Cu powder.

**Table 2:** Roughness of electrically conductive films containing untreated Ag/Cu and alkanethiol-modified Ag/Cu powders

Sample	Ra (µm)	Rz (µm)
Ag/Cu	2.043 ± 0.15	13.459 ± 1.54
Ag/Cu-C_4_	0.719 ± 0.17	8.408 ± 1.13
Ag/Cu-C_8_	0.760 ± 0.13	8.608 ± 1.01
Ag/Cu-C_12_	0.631 ± 0.10	7.898 ± 0.47

This suggests that the alkanethiol modification of Ag/Cu powders improves their compatibility with the epoxy resin to some extent. However, no significant correlation between the surface roughness of the film and the length of the alkyl chain of the alkanethiol-modified Ag/Cu powders is evident.

**Fig. 5:**
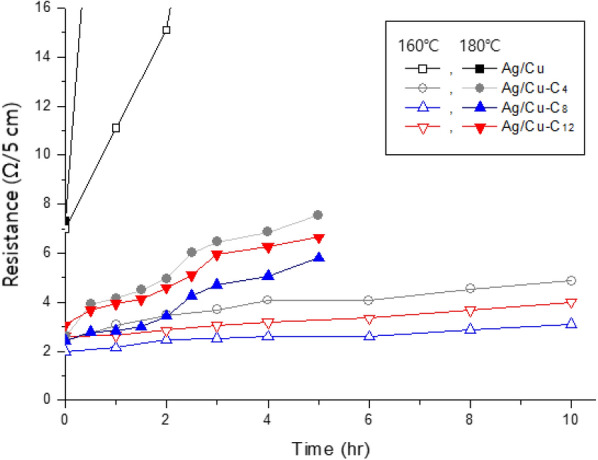
Resistance of electrically conductive films containing untreated Ag/Cu and alkanethiol-modified Ag/Cu powders incubated at 160 and 180 °C, respectively

We also investigated the thermal stability of the electrically conductive films. As established by the TGA of the untreated Ag/Cu powder and previously reported studies [17, 29], the oxidation of Ag/Cu powder commences at a temperature of approximately 170–175 °C. Accordingly, the change in the linear resistance of each electrically conductive film over time was monitored in an oven at temperatures of 160 and 180 °C, respectively; the results are plotted in Fig. 5.

**Fig. 6:**
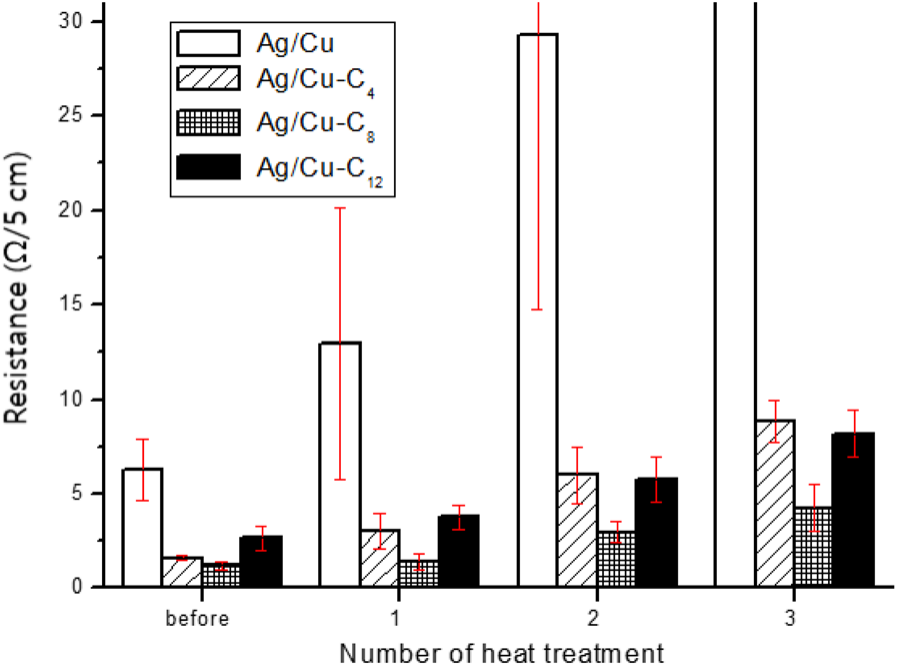
Resistance of electrically conductive films containing untreated Ag/Cu and alkanethiol-modified Ag/Cu after successive heat treatments at 260 °C

The electrically conductive film containing untreated Ag/Cu powder demonstrates much higher electrical resistance than those containing alkanethiol-modified Ag/Cu powders. The high initial resistance is attributed to the surface oxidation of the Au/Cu powder that occurred during the 1-h curing process at 160 °C before thermal stability test. As shown in Fig. 5, the resistance of the film containing untreated Ag/Cu powder increases rapidly at 160 °C over time, and even more rapidly at 180 °C. In contrast, the resistance of electrically conductive films containing alkanethiol-modified Ag/Cu powders remains relatively stable at 160 °C, their resistance increases by less than 50 % after 10 h of incubation at 160 °C. However, the resistance of the films increases more rapidly at 180 °C; the measurements had to be halted after 5 h owing the twisting of the films. In addition, while there is no significant difference in the resistance behaviors of the films containing alkanethiol-modified Ag/Cu powders, their resistance increases in terms of alkyl-chain length in the order: C_8_ < C_12_ < C_4_. The above results show that the thermal stability of the Ag/Cu powder is significantly improved by the adsorbed alkanethiols. Therefore, soldering heat resistance of the films shown in Fig. 6 were evaluated according to the method described in the experimental section that simulates soldering conditions.

The test prescribed involves processing each film at 260 °C for 10 s and then measuring the linear resistance at room temperature, the heat treatment and measurement process is repeated three times. The resistance of the film containing untreated Ag/Cu powder is relatively high after the first heat treatment, while the resistance of the films containing alkanethiol-modified Ag/Cu powders is relatively stable, indicating that the thermal stability of the films is improved by the modification of the Ag/Cu powders with alkanethiols. The effect of the alkyl-chain length of the alkanethiol-modified Ag/Cu powders is also evident, i.e., the film containing the Ag/Cu powder modified with octanethiol (Ag/Cu-C_8_) is the most stable film. In the case of the film containing butanethiol-modified Ag/Cu powder (Ag/Cu-C_4_), the increase in its resistance after each heat treatment is greater than the corresponding increase observed in the resistance of the film containing dodecanethiol-modified Ag/Cu powder (Ag/Cu-C_12_). These results are judged to indicate efficient thermal stability; although, the longer the alkyl chain of the alkanethiol, the lower the electrical properties of the alkanethiol-modified Ag/Cu powder. In practice, the rate at which the resistance of the film increases (R3/R0), i.e., the linear resistance after three heat treatments (R3) divided by the original linear resistance (R0), is found to be 5.57, 3.54, and 3.08 for the Ag/Cu-C_4_, Ag/Cu-C_8_, and Ag/Cu-C_12_ films, respectively, indicating that the longer the alkyl chain, the smaller the increase in resistance induced by the heat treatment. In other words, the longer the alkyl chain of the alkanethiol-modified Ag/Cu powder, the higher the resistance of the film due to the greater amount of organic matter surrounding the Ag/Cu powder particles; however, the longer the alkyl chain of the alkanethiol-modified Ag/Cu powder, the lower its vulnerability to oxidation and the smaller the increase in the resistance of the film caused by heat treatment. These effects combine to produce the lowest resistance in the Ag/Cu-C_8_ film after three heat treatments.

## Conclusions

In order to improve the thermal stability of dendritic Ag/Cu powders, the surfaces of the Ag/Cu powders were modified with alkanethiols featuring alkyl chains of different lengths. These powders were used to fabricate electrically conductive films in combination with an epoxy resin. The thermal stability of the conductive films (at 160 and 180 °C) was investigated and the films were also subjected to a thermal stability test(three successive heat treatments at 260 °C for 10 s each). The results confirm that the modification of Ag/Cu powders with alkanethiols remarkably improve their thermal stability. The thermal stability of the films containing alkanethiol-modified Ag/Cu powders does not vary significantly in terms of alkyl-chain length. The longer the alkyl-chain of the alkanethiol-modified Ag/Cu powder, the higher the initial resistance of the associated electrically conductive film. However, the increase in the resistance of the films containing alkanethiol-modified Ag/Cu powders due to heat treatment is relatively small. As a result, the best electrical characteristics are demonstrated by the film containing octanethiol-modified Ag/Cu powder (Ag/Cu-C_8_). The results of this study suggest that Ag/Cu powders are promising fillers for electrically conductive adhesives.

## Data Availability

Not applicable.

## Abbreviations

SEM-EDS: Scanning electron microscopy with energy dispersive spectroscopy
XPS: X-ray photoelectron spectroscopy
TGA: Thermogravimetric analysis
Ra: Arithmetic mean roughness
Rz: Ten-point mean roughness
